# Single-cell RNA sequencing reveals cell landscape following antimony exposure during spermatogenesis in *Drosophila* testes

**DOI:** 10.1038/s41420-023-01391-4

**Published:** 2023-03-09

**Authors:** Jun Yu, Yangbo Fu, Zhiran Li, Qiuru Huang, Juan Tang, Chi Sun, Peiyao Zhou, Lei He, Feiteng Sun, Xinmeng Cheng, Li Ji, Hao Yu, Yi Shi, Zhifeng Gu, Fei Sun, Xinyuan Zhao

**Affiliations:** 1 Institute of Reproductive Medicine, Medical School of Nantong University, Nantong University, Nantong, 226001 China; 2 Department of Occupational Medicine and Environmental Toxicology, Nantong Key Laboratory of Environmental Toxicology, School of Public Health, Nantong University, Nantong, 226019 China; 3 Department of Geriatrics, Affiliated Hospital of Nantong University, Nantong University, Nantong, 226001 China; 4 Department of Rheumatology, Affiliated Hospital of Nantong University, Nantong University, Nantong, 226001 China

**Keywords:** Cell lineage, Cell growth

## Abstract

Antimony (Sb), is thought to induce testicular toxicity, although this remains controversial. This study investigated the effects of Sb exposure during spermatogenesis in the *Drosophila* testis and the underlying transcriptional regulatory mechanism at single-cell resolution. Firstly, we found that flies exposed to Sb for 10 days led to dose-dependent reproductive toxicity during spermatogenesis. Protein expression and RNA levels were measured by immunofluorescence and quantitative real-time PCR (qRT-PCR). Single-cell RNA sequencing (scRNA-seq) was performed to characterize testicular cell composition and identify the transcriptional regulatory network after Sb exposure in *Drosophila* testes. scRNA-seq analysis revealed that Sb exposure influenced various testicular cell populations, especially in GSCs_to_Early_Spermatogonia and Spermatids clusters. Importantly, carbon metabolism was involved in GSCs/early spermatogonia maintenance and positively related with SCP-Containing Proteins, S-LAPs, and Mst84D signatures. Moreover, Seminal Fluid Proteins, Mst57D, and Serpin signatures were highly positively correlated with spermatid maturation. Pseudotime trajectory analysis revealed three novel states for the complexity of germ cell differentiation, and many novel genes (e.g., Dup98B) were found to be expressed in state-biased manners during spermatogenesis. Collectively, this study indicates that Sb exposure negatively impacts GSC maintenance and spermatid elongation, damaging spermatogenesis homeostasis via multiple signatures in *Drosophila* testes and therefore supporting Sb-mediated testicular toxicity.

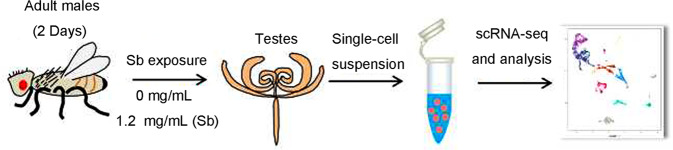

## Introduction

As a silvery white metalloid of medium hardness, antimony (Sb) is generally combined with other metals to create hardened alloys, followed by industrial applications and commodity consumption, and finally leading to environmental discharge [1]. In addition to human activities, Sb in the environment can come from natural processes, such as ore weathering or volcanic activity [2]. Once it’s released into the environment, Sb is found in a variety of environmental media, including water, soil, and sediment, and even in atmospheric aerosols [3]. As a result, Sb can enter the human body through the respiratory tract, digestive tract, and skin. Oral exposure to Sb is also common due to its emetic properties [4]. Antimonials have been used to treat two parasitic diseases, schistosomiasis and leishmaniasis. Based on epidemiological studies, Sb levels in the urine or blood may be positively associated with adverse population health outcomes [5, 6], suggesting that exposure-mediated health risks should be investigated.

The primary toxic effect of Sb is cardiotoxicity when used in clinical therapeutic preparations [7]. On the other hand, occupational exposure to Sb leads to respiratory irritation or pneumoconiosis [8]. Our previous studies first tried to elucidate the neurotoxic effects and mechanisms of Sb [9, 10]. Male infertility is an important issue and has been associated with environmental pollution, including that of heavy metals [11]. Wu et al. assessed the effects of Sb exposure on reproductive organs in adult male mice and found that parameters of sperm quality, such as sperm count and the testis coefficient, were dramatically decreased [12]. However, an earlier study showed that Sb potassium tartrate exposure administered by gavage neither decreased testis or accessory sex organ weight nor impacted sperm parameters in rats or mice [13]. Taken together, Sb exposure-triggered testicular toxicity remains unclear and further research is urgently needed.

*Drosophila* has been used as an animal model for more than 100 years; discoveries in fruit flies have greatly contributed to our understanding of spermatogenesis and pollutant-mediated toxicity [14–16]. Spermatogenesis is a highly conserved process in *Drosophila* and mammalian testes and is well-understood from an anatomical and histological perspective [17, 18], but the underlying regulatory foundations between environmental pollution and genetic heredity are poorly understood. In *Drosophila*, the testis contains a well-structured microenvironment, the stem cell niche, which is comprised of terminally differentiated hub cells, germline stem cells (GSCs), and somatic cyst stem cells (CySCs) [19]. Hub cells maintain the self-renewal and differentiation of the two other types of stem cells [20, 21]. Interestingly, germ cells occupy the majority of testicular components, and their maintenance and differentiation are strictly controlled by functional signals that originate from somatic cells in *Drosophila* testes [22, 23]. CySCs differentiate into cyst cells and provide the environment necessary to trigger germ cell growth and differentiation via non-autonomous effects [24]. With the encapsulation of somatic cyst cells, GSCs produce gonialblasts (GBs) and undergo transit amplification (TA) with four rounds of mitosis and then differentiate into spermatocytes and spermatids, ultimately forming mature sperm [25].

Therefore, *Drosophila* is a suitable model system to assess cytotoxic effects induced by environmental pollutant exposure [26]. This study aims to explore the testicular toxicity of Sb with regard to GSC maintenance, germ cell differentiation, and spermatid deformation. In particular, we apply single-cell RNA sequencing (scRNA-seq) to comprehensively characterize the transcriptional regulatory network of different cell populations in *Drosophila* testes following Sb exposure. Our data provide meaningful information for Sb-mediated testicular toxicity identification.

## Results

### Cellular effects of Sb exposure in *Drosophila* testes

**Fig. 1:**
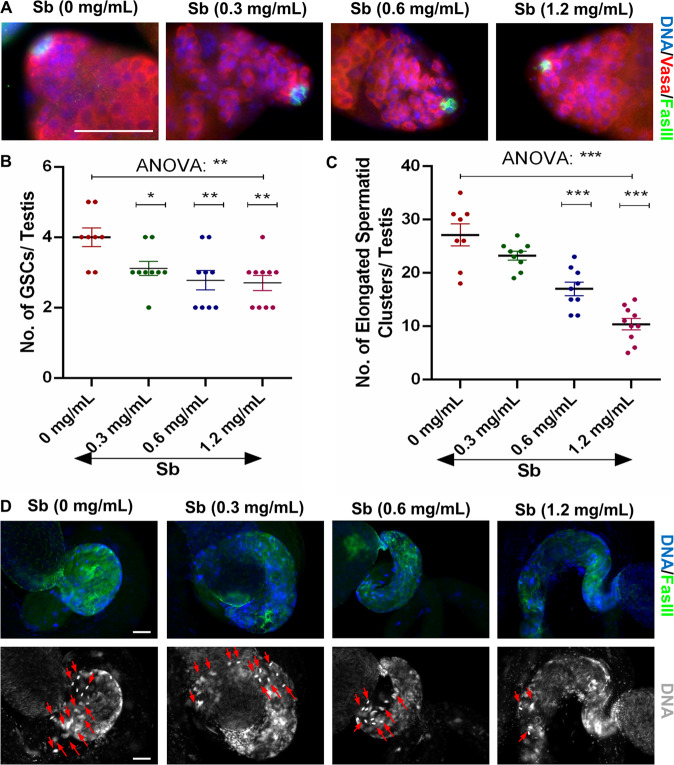
Sb exposure injures the maintenance of GSCs and elongated spermatid clusters. **A** Immunostaining of Vasa (red) and FasIII (green) at the apex of testis under Sb exposure at 0 mg/mL, 0.3 mg/mL, 0.6 mg/mL and 1.2 mg/mL. **B** The number of GSCs per testis. **C** The number of elongated spermatid clusters per testis. **D** FasIII (green) immunostaining at the tail of testis under Sb exposure at 0 mg/mL, 0.3 mg/mL, 0.6 mg/mL and 1.2 mg/mL. DNA was stained with Hoechst33342 (blue or grey). Representative clusters of elongated spermatids were shown with red arrows. *P < 0.05, **P < 0.01, ***P < 0.001, scale bar: 50 μm.

The *Drosophila* testis has been widely used as a model for male reproductive toxicity due to clear observations of different cell structures during spermatogenesis. To investigate the toxicological effects of Sb exposure in *Drosophila* testes, we examined the numbers of GSCs and elongated spermatid clusters in *Drosophila* testes following Sb exposure at 0, 0.3, 0.6, and 1.2 mg/mL.Vasa was used to label germ cells, and De-cad marked somatic hub cells and cyst cells in testes (Fig. S1). Importantly, GSCs could be identified by Vasa-positive germ cells directly adjacent to hub cells at the apex of the testis, while elongated spermatid nuclei gathered in a cluster and were highly agglutinated at the tail of the testis. Compared to control testes, Sb exposure at 0.3, 0.6, or 1.2 mg/mL dramatically reduced the number of GSCs in a dose-dependent manner (Fig. 1A, B). Whole mount staining of Vasa for 0 mg/mL Sb-treated testes (control group) and 1.2 mg/mL Sb-treated testes (Sb group) demonstrated that partial germ cell cysts were lost after Sb-treated (1.2 mg/mL) in testes (Fig. S2A). Previous study demonstrated that A-type Lamin C (LamC) was dominated expressed in spermatocytes [27]. Despite the fact that the property of missing germ cell cysts were spermatocytes, whole mount testicular LamC staining (Fig. S2B) also indicated that spermatocyte populations were not seriously damaged and the remaining spermatocytes could continue to differentiate after Sb exposure (1.2 mg/mL).

We next performed phase-contrast visualization of squashed adult testes to identify different stages of germ cells during spermatogenesis. Different stages of testicular germ cells including spermatogonia (Spg), spermatocytes (Spc), round spermatids (Round Spd), elongated spermatids (Elongated Spd) and mature sperm (Sperm) could be found in testes of both control and Sb groups. Importantly, we also identified accumulations of abnormal elongated spermatids and sperm after Sb exposure (1.2 mg/mL), while they were not found in control group (Fig. S2C).

DNA staining highly condensed at the early-stage nuclei of germ cells and the nuclei of clusters of elongated spermatozoa in the *Drosophila* testis [16, 23]. Based on the above observation, we next counted the number of elongated spermatid clusters and found a significant and dose-dependent decrease (Fig. 1C, D), indicating that Sb exposure participated in differentiation and injured elongated spermatozoon formation in *Drosophila* testes. We also stained with Dcp-1 and found that Sb exposure (1.2 mg/mL) dramatically induced apoptosis in elongated spermatid clusters (Fig. S2D). After Sb exposure (1.2 mg/mL), we also found that males reduced their fertility ability (male fertility rate: 73.33%, *n* = 30) when compared with control males (male fertility rate: 91.67%, *n* = 12) (Fig. S2E). Thus, Sb exposure in *Drosophila* induced reproductive toxicity during spermatogenesis.

### scRNA-seq analysis identified comprehensive cell compositions in testes

**Fig. 2:**
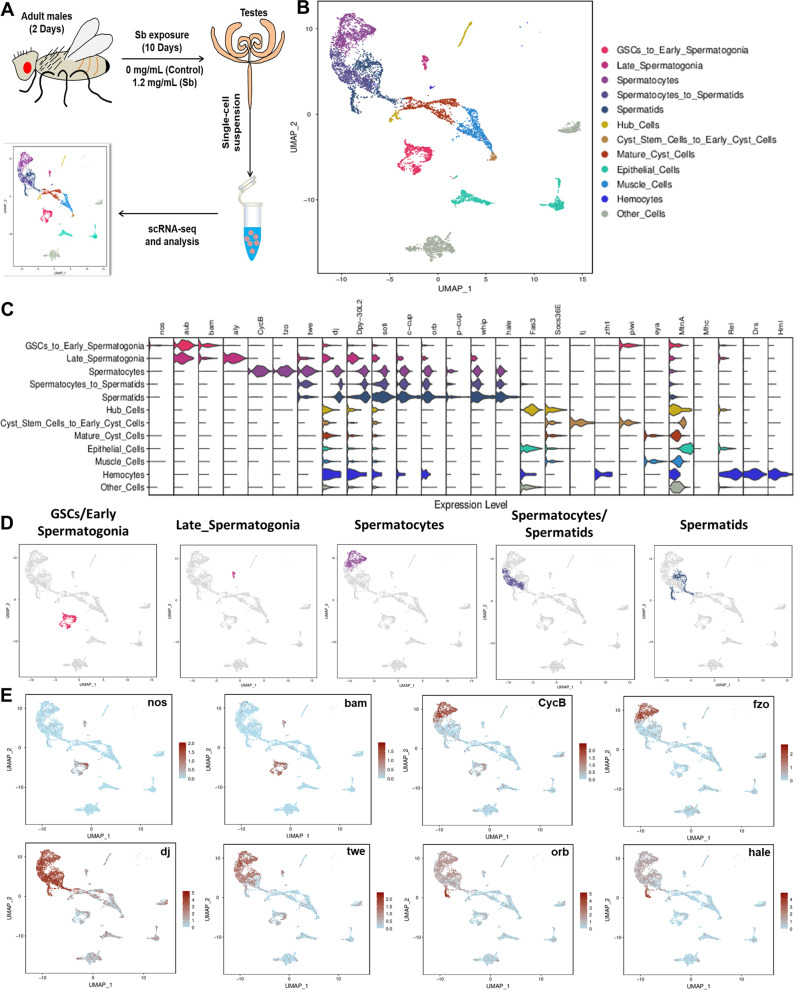
Identification of testicular cell clusters by scRNA-seq. **A** An illustrated flow chart of scRNA-seq in the *Drosophila* testis. **B** Annotations of testicular cell clusters via UMAP visualization. **C** The violin plot views of expression patterns for marker genes in the *Drosophila* testis. **D** UMAP visualizations of germ cells at different stages. **E** UMAP visualizations of representative marker genes.

To classify the cell types affected by Sb exposure in *Drosophila* testes, we performed scRNA-seq on the cells isolated from fresh testes with or without Sb treatment. A cell suspension was made into a library and sequenced (Fig. 2A). We obtained 10,715 testicular cells; low-quality cells were filtered out. After filtering, 9180 high quality cells were retained for downstream analyses with an average of 10,821.5 median UMIs and 2058 median genes per cell. The dimensionality of the gene/cell expression matrix was reduced to two primary axes and visualized through UMAP in Seurat, gathering similar cells into clusters. We annotated likely cell types for each cluster based on the expression pattern of published marker genes, and these clusters could be annotated into 12 cell populations that covered almost all testicular cell types in *Drosophila* (Fig. 2B and Table S3). Importantly, many cell types were identified by multiple marker genes and their expression patterns are shown in Fig. 2C. During spermatogenesis in *Drosophila*, different stages of germ cells could be clearly distinguished by various marker genes (Fig. 2D, E). For instance, nanos (*nos*) is a key marker of GSCs and early germ cells, and bag of marbles (*bam*) is specifically expressed in spermatogonia [28, 29]. Moreover, Cyclin B (*CycB*) is mainly expressed in spermatocytes, while fuzzy onions (*fzo*) are highly enriched in spermatocytes and early spermatids [30, 31]. We also used several classical markers to identify spermatids, including don juan (*dj*), twine (*twe*), oo18 RNA-binding protein (*orb*), calcutta cup (*c-cup*), and presidents-cup (*p-cup*) [18, 32]. Expression patterns of representative marker genes are visualized by UMAP in Fig. 2E.

### Cell population and changes in transcription following Sb exposure

**Fig. 3:**
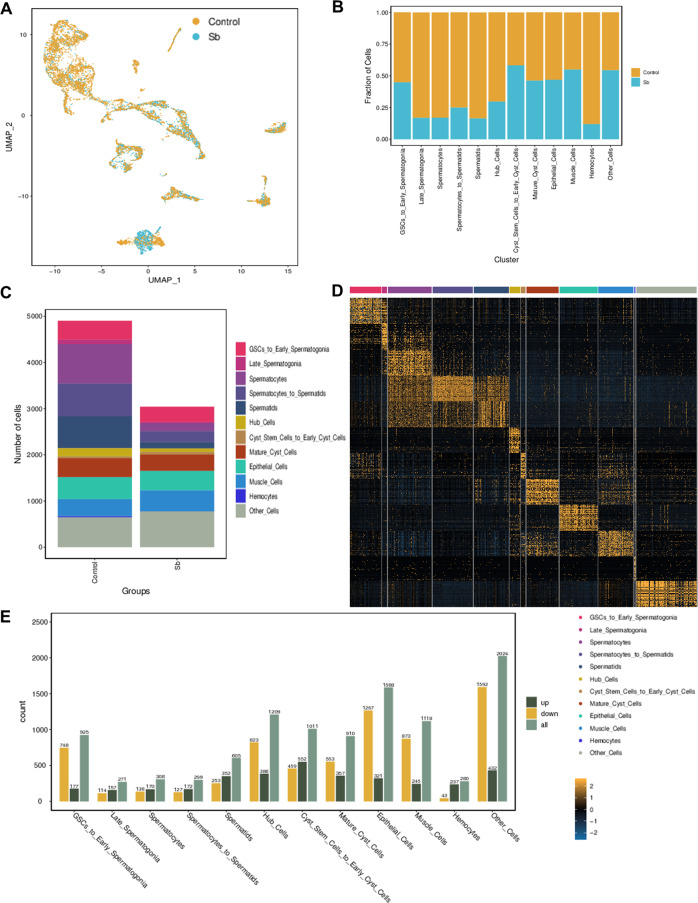
Features of testicular cell populations in *Drosophila*. **A** UMAP visualization of testicular cells in each group. **B** Fraction of testicular cells in different cell clusters of control and Sb groups. **C** Cellular component analysis in control and Sb groups. **D** The heatmap of the most abundantly expressed genes in different cell clusters. **E** Analysis of DEGs in different cell clusters.

Distributions of cell populations in the control and Sb-treated groups were merged by UMAP (Fig. 3A). Overall, both the fraction and number of cell populations were dramatically reduced following Sb exposure (Fig. 3B, C). These cell fate changes appeared most significantly in germ cells, revealing reproductive toxicity of Sb exposure during spermatogenesis. The top 50 highly expressed genes were observed in each cell type in *Drosophila* testes (Fig. 3D and Table S4). Dynamic changes in the gene expression pattern and the number of DEGs in different cell populations are shown in Fig. 3E. Since phenotypes of Sb exposure for spermatogenesis were identified in GSCs and spermatids, the following analysis mainly focused on the GSCs_to_Early_Spermatogonia and Spermatids clusters. DEGs in germ cells were dramatically increased in the GSCs_to_Early_Spermatogonia and Spermatids clusters, with 925 (177 upregulated and 748 downregulated) and 605 (352 upregulated and 253 downregulated) DEGs in these cell clusters, respectively.

### Transcriptional regulation of GSCs/early spermatogonia maintenance following Sb exposure

**Fig. 4:**
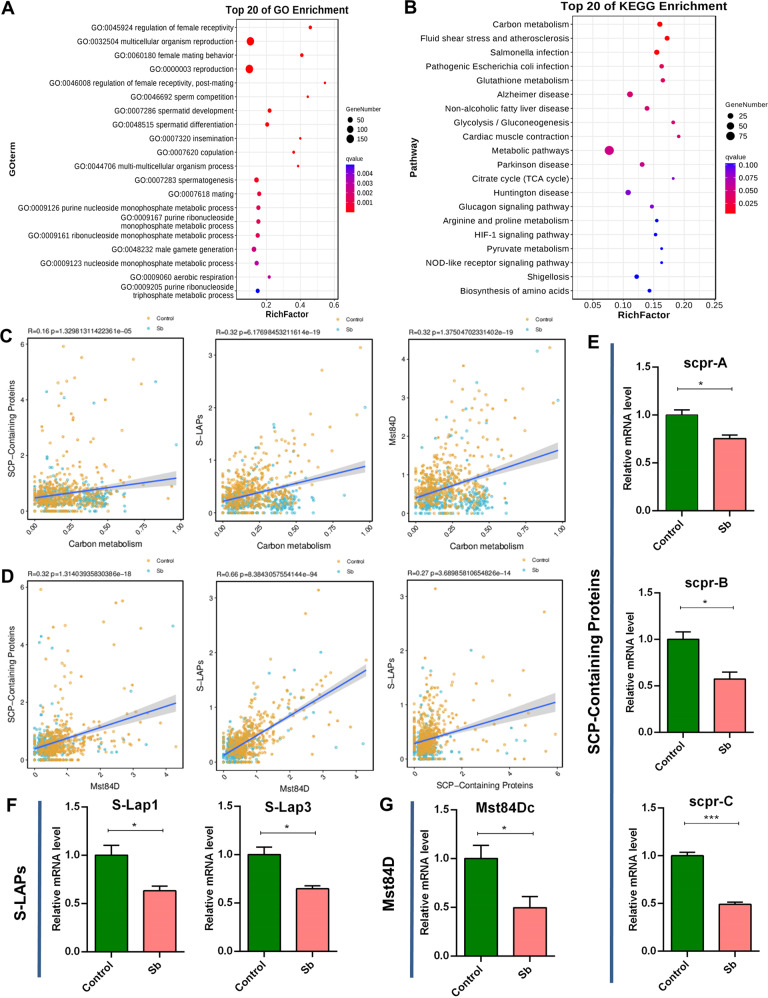
Referred signatures for DEGs in mitotic germ cells. **A** Top 20 of GO enrichment for DEGs in GSCs_to_Early_Spermatogonia cell cluster. **B** Top 20 of KEGG enrichment for DEGs in GSCs_to_Early_Spermatogonia cell cluster. **C** Correlation analysis of carbon metabolism with SCP-Containing Proteins, S-LAPs, and Mst84D signatures in GSCs_to_Early_Spermatogonia cluster, respectively. **D** Correlation analysis among SCP-Containing Proteins, S-LAPs, and Mst84D signatures in GSCs_to_Early_Spermatogonia cluster. **E** qRT-PCR analysis of representative DEGs for SCP-Containing Proteins signature in testes. **F** qRT-PCR analysis of representative DEGs for S-LAPs signature in testes. **G** qRT-PCR analysis of representative DEG for Mst84D signature in testes. *P < 0.05, ***P < 0.001.

To underline Sb-mediated molecular regulation of early stage germ cells, we performed GO enrichment for DEGs in GSCs_to_Early_Spermatogonia cell types; enriched terms were related with multicellular organism reproduction and spermatogenesis (Fig. 4A). Parallel KEGG pathway analysis showed that various metabolic pathways, including carbon metabolism, were highly enriched in the interactions between Sb exposure and genetic heredity for GSCs/early spermatogonia maintenance (Fig. 4B).

Meanwhile, we also noticed that the expression trends of several family members (also named ‘signatures’ in this study) showed high consistency after Sb exposure in *Drosophila* testes. We identified several signatures (SCP-Containing Proteins, S-LAPs, and Mst84D signatures) that were altered dramatically following Sb exposure, and investigated correlations of these signatures with carbon metabolism pathway in corresponding testicular cell populations. Our results showed that carbon metabolism exhibited a positive correlation coefficient with SCP-Containing Proteins, S-LAPs, and Mst84D signatures (Fig. 4C). Further correlation analysis indicated positive correlations between any two of the three signatures: (1) SCP-Containing Proteins vs. Mst84D, (2) S-LAPs vs. Mst84D, and (3) SCP-Containing Proteins vs. S-LAPs (Fig. 4D).

We next examined the relative mRNA levels of key factors in correlative signatures in *Drosophila* testes, and found that representative DEGs of carbon metabolism related factors (*CG7059*, *CG32026* and *CG9314*), SCP-Containing Proteins [SCP-containing protein A (*scpr-A*), SCP-containing protein B (*scpr-B*), and SCP-containing protein C (*scpr-C*)], S-LAPs [Sperm-Leucylaminopeptidase 1 (*S-Lap1*) and Sperm-Leucylaminopeptidase 3 (*S-Lap3*)], and Mst84D [Male-specific RNA 84Dc (*Mst84Dc*)] were dramatically decreased in testes exposed to Sb compared with control testes (Fig. 4E–G and Fig. S3). Taken together, these data provided novel transcriptional regulatory signals in GSCs_to_Early_Spermatogonia cell populations, contributing to the understanding of Sb exposure mediated GSCs/early spermatogonia maintenance.

### Spermatid cell profiling revealed molecular signatures following testicular Sb exposure

**Fig. 5:**
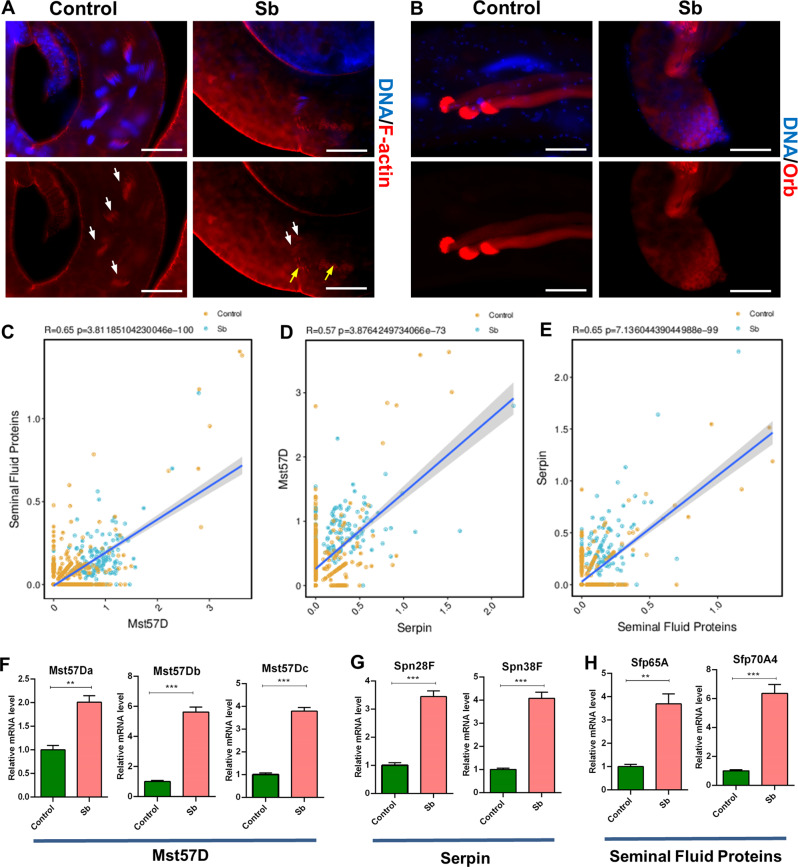
Relevant molecular signaling in spermatid development. **A** F-actin staining for the investigation of IC structure in control and Sb testes. Representative ICs were shown with white arrows and abnormal IC structures were labelled with yellow arrows. **B** Immunostaining of Orb (red) mainly to label elongated spermatids in control and Sb testes. **C** Correlation analysis between Seminal Fluid Proteins and Mst57D signatures related in Spermatids cluster. **D** Correlation analysis between Mst57D and Serpin signatures in Spermatids cluster. **E** Correlation analysis between Serpin and Seminal Fluid Proteins signatures in Spermatids cluster. **F** qRT-PCR analysis of representative DEGs for Mst57D signature in testes. **G** qRT-PCR analysis of representative DEGs for Serpin signature in testes. **H** qRT-PCR analysis of representative DEGs for Seminal Fluid Proteins signature in testes. DNA was stained with Hoechst33342 (blue). **P < 0.01, ***P < 0.001, scale bar: 50 μm.

To further evaluate testicular toxicity following Sb exposure during late stage spermatogenesis, we stained with several spermatid markers. F-actin marks the individualization complex (IC), a structure essential for spermatid individualization. After Sb exposure, the IC was dramatically reduced at the tail of testes and the remnant IC structure was severely damaged (Fig. 5A), leading to the differentiation defects of elongated spermatids. Orb was used as a classical marker with highly enriched expression level in elongated spermatids [23, 32]. Our results showed that the expression pattern of Orb protein was disturbed at late stage of spermatogenesis in control and Sb-exposed testes (Fig. 5B). Combined with our scRNA-seq data, we also identified Seminal Fluid Proteins, Mst57D, and Serpin signatures related to testicular Sb exposure in the Spermatids cell type. Correlation analysis in spermatid populations showed that DEGs in Seminal Fluid Proteins exhibited a positive correlation coefficient with DEGs in Mst57D (Fig. 5C) and DEGs in Mst57D exhibited a positive correlation coefficient with DEGs in Serpin (Fig. 5D); DEGs in Serpin also exhibited a positive correlation coefficient with DEGs in Seminal Fluid Proteins (Fig. 5E). We also noticed that DEGs in these signatures showed consistent expression trends in most testicular cell populations. Our qRT-PCR results showed that representative DEGs in Mst57D [Male-specific RNA 57 Da (*Mst57Da*), Male-specific RNA 57Db (*Mst57Db*), and Male-specific RNA 57Dc (*Mst57Dc*)], Serpin [Serpin 28 F (*Spn28F*) and Serpin 38 F (*Spn38F*)], and Seminal Fluid Proteins [Seminal fluid protein 65 A (*Sfp65A*) and Seminal fluid protein 70A4 (*Sfp70A4*)] signatures were significantly upregulated in the Sb-exposed group compared with the control group (Fig. 5F–H). The above data identified three major signatures in *Drosophila* testes following Sb exposure during spermatid development.

### Pseudotime trajectory analysis demonstrated three novel states for Sb exposure during spermatogenesis

**Fig. 6:**
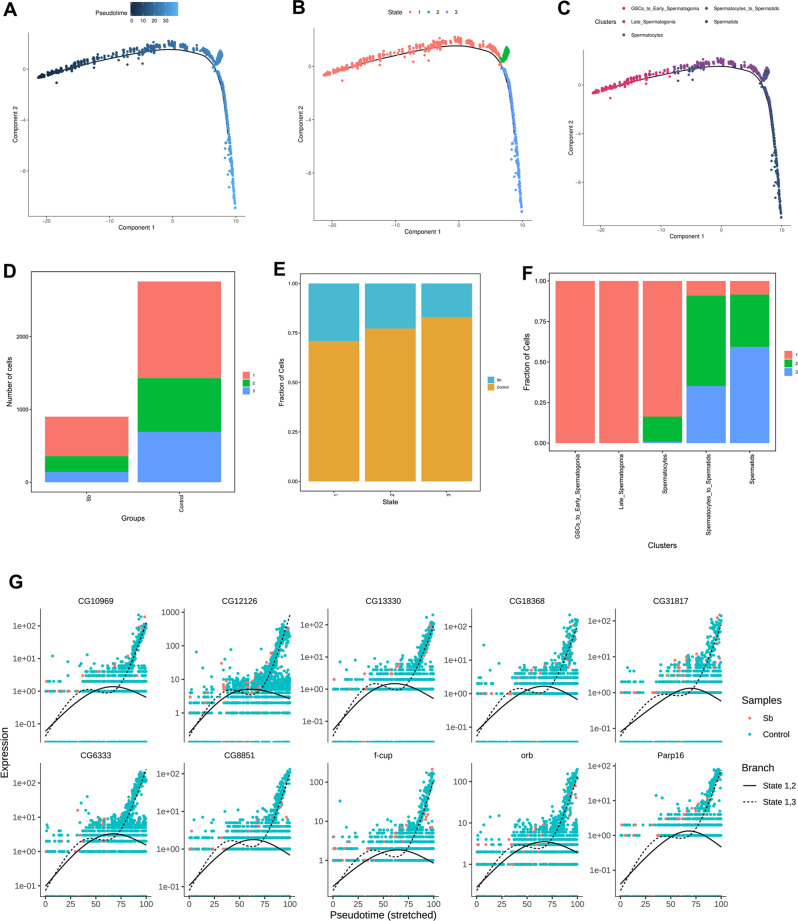
Pseudotime trajectory analysis for testicular germ cell complexity during spermatogenesis. **A** Analysis of germ cells visualized by pseudotime trajectory plot. **B** Pseudotime trajectory analysis of germ cells colored by different states. **C** Pseudotime trajectory analysis of germ cells colored by different cell populations. **D** The number of testicular cells in each state of control and Sb groups. **E** The fraction of testicular cells in each state of control and Sb groups. **F** Cellular component analysis in each state during spermatogenesis. **G** Spline plots of representative gene expression dynamics for state 1, 2 branch and state 1, 3 branch in germ cell populations.

To further investigate the germ cell features in *Drosophila* testes following Sb exposure, we conducted a pseudotime trajectory analysis of germ cell maturation during spermatogenesis (Fig. 6A), and identified three novel states in *Drosophila* testes (Fig. 6B). By analyzing the cellular components in each state, we showed that the germ cells were consistent with differentiative stages (Fig. 6C). It is worth noting that both the number and fraction of germ cells in each state were dramatically reduced following Sb exposure (Fig. 6D, E). Importantly, germ cells in state 1 were composed of pre-meiotic GSCs/early spermatogonia and meiotic spermatocytes, while germ cells in state 2 and state 3 trended to be clustered by meiotic spermatocytes and post-meiotic spermatids (Fig. 6F). Consistent with the expression patterns of different cell populations during spermatogenesis, a large number of genes were specifically expressed in a state-biased manner. To further distinguish gene regulation in state 1, branch 2 and state 1, branch 3, spline plots were used to demonstrate the expression dynamics of the two branches. The resultant pseudotime analysis indicated that state 1, branch 3 directs to germ cell populations with high-level expression of identified novel factors and marker genes, while these genes are gradually decreased in state 1, branch 2 (Fig. 6G).

**Fig. 7:**
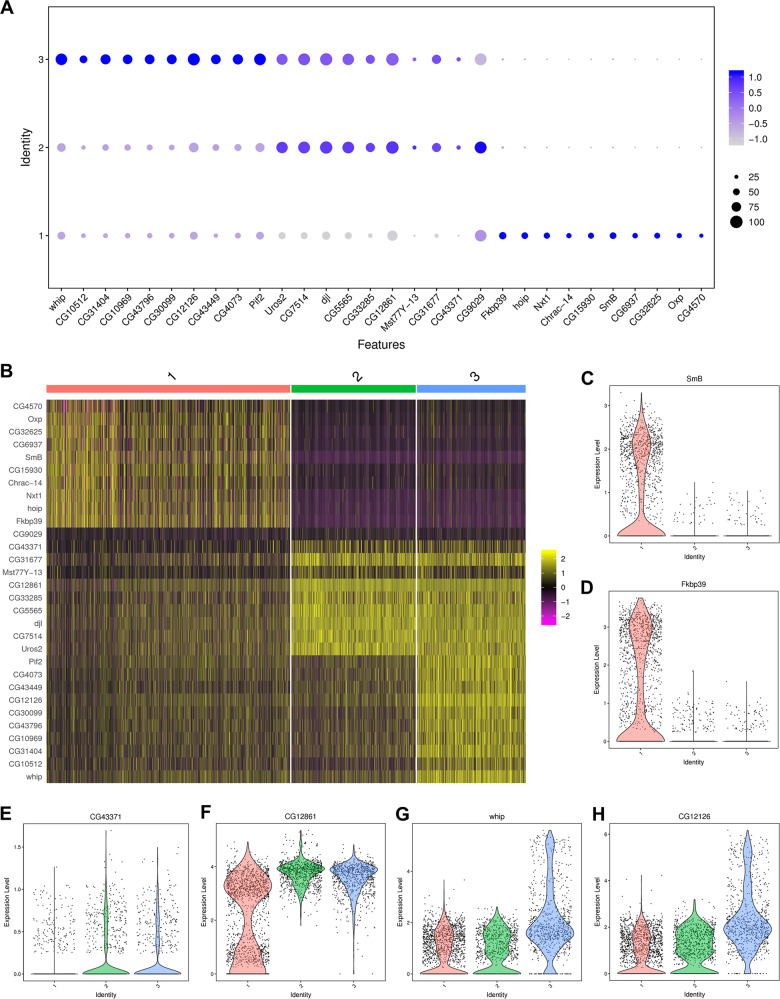
Characteristic of three novel states during spermatogenesis. **A** The dot plot view of representative highly expressed genes in each state of germ cell populations. **B** The heatmap view of representative highly expressed genes in each state of germ cell populations. **C** The violin plot view of *SmB* in three states of germ cell populations. **D** The violin plot view of *Fkbp39* in three states of germ cell populations. **E** The violin plot view of *CG43371* in three states of germ cell populations. **F** The violin plot view of *CG12861* in three states of germ cell populations. **G** The violin plot view of *whip* in three states of germ cell populations. **H** The violin plot view of *CG12126* in three states of germ cell populations.

To further analyze the characteristics of the three states in *Drosophila* testes following Sb treatment, dot plot and heatmap views of the most highly expressed genes are shown in Fig. 7A and B, respectively. Three major expression patterns were found: genes such as those encoding the small ribonucleoprotein particle protein SmB (*SmB*) and FK506-binding protein 39 kDa (*Fkbp39*) were specifically expressed in state 1 (Fig. 7C, D), genes such as *CG43371* and *CG12861* were predominantly expressed in state 2 and state 3 (Fig. 7E, F), and genes such as whipple (*whip*) and *CG12126* were highly enriched in state 3 (Fig. 7G, H). Among them, SmB formed the heterodimeric sub-complex with SmD3 production, and previous studies have indicated that both SmB and SmD3 were essential for GSCs homeostasis in *Drosophila* testes [22, 33]. Whip was dominant expressed in testes and mainly used as a marker for elongation stage spermatids [34].

**Fig. 8:**
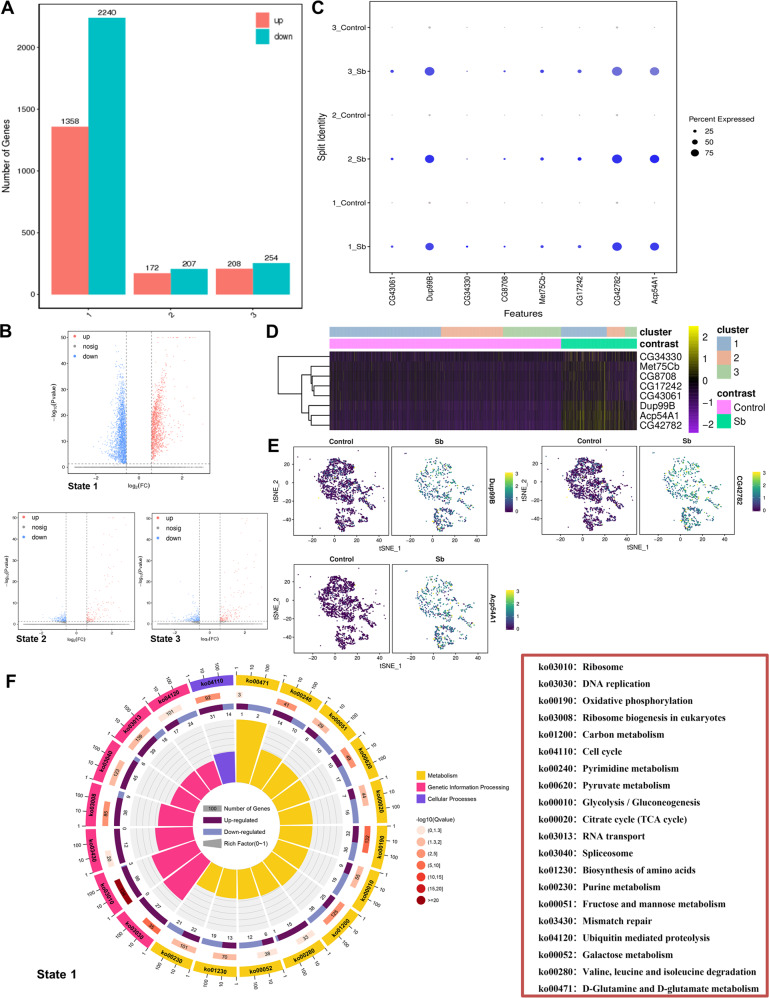
Analysis of Sb exposure mediated DEGs in three novel states of germ cell populations. **A** The number of DEGs in three states of germ cell populations between control and Sb groups. **B** The volcano plot views of DEGs in three states between two groups. **C** The dot plot view of representative enriched genes which are upregulated in all three states of germ cell populations. **D** The heatmap view of representative enriched genes in three states of germ cell populations. **E** tSNE visualizations of *Dup99B*, *CG42782* and *Acp54A1* genes in control and Sb groups. **F** The KEGG analysis for DEGs in state 1 germ cell populations.

Next, we analyzed dynamic changes in the gene expression pattern in each testis state in control and Sb-treated *Drosophila*, and identified 3598 (1358 upregulated and 2240 downregulated) DEGs in state 1, 379 (172 upregulated and 207 downregulated) DEGs in state 2, and 462 (208 upregulated and 254 downregulated) DEGs in state 3 (Fig. 8A). Furthermore, volcano plots showed distinct differences in the DEGs in each state of germ cell populations between the two groups (Fig. 8B). We also identified several enriched genes that were upregulated following Sb exposure in all states during germ cell differentiation (Fig. 8C). A heatmap was used to show the expression pattern of eight upregulated marker genes [*CG34330*, *Met75Cb*, *CG8708*, *CG17242*, *CG43061*, Ductus ejaculatorius peptide 99B (*Dup99B*), Accessory gland protein 54A1 (*Acp54A1*), and *CG42782*] that were identified in all differentiated states in control and Sb-exposed germ cells (Fig. 8D). Among them, *Dup99B*, *CG42782*, and *Acp54A1* were found to be representative and significantly enriched genes; these genes were visualized by t-SNE maps and dramatically upregulated following Sb exposure (Fig. 8E). Since we identified a large number of DEGs in state 1, we performed a KEGG analysis of state 1 germ cells. We found that the enriched cellular processes were involved in cell growth and death, and the main genetic processing focused on the synthesis and degradation of proteins (Fig. 8F). We also noticed that many metabolic processes, including carbon metabolism, oxidative phosphorylation, glycolysis/gluconeogenesis, and the citrate cycle, participated in the differentiation of state 1 germ cells (Fig. 8F). These changes in gene expression and enrichment analysis revealed that the germ cells in the different states went through diversified signals to regulate spermatogenesis following Sb exposure.

## Discussion

*Drosophila* has emerged as an important model for understanding conserved biological mechanisms, drawing support from the availability of convenient genetic manipulations [35–37]. scRNA-seq has provided insight into the genetic regulation of different cell populations in multiple species [38, 39]. Combined with powerful genetic tools in the fly, scRNA-seq can be used to identify novel cell states and functions at single-cell resolution [40]. Recent single-cell transcriptomic atlases have characterized novel cell populations involved in different biological events, uncovering transcriptional regulation or signaling pathways in *Drosophila* [41–44]. Li et al. constructed a large scale adult fruit fly atlas via single-nucleus transcriptomics, which has become a valuable resource for the *Drosophila* community as it has provided novel insight into rare cell types and tissue-specific sub-types [34]. In the field of environmental toxicology, *Drosophila* is known as an alternate animal model to explore stimuli-mediated male reproductive toxicity [45].

Accumulating evidence has revealed cellular networks at the single-cell level in both mouse and human testicles [46–48]. In *Drosophila*, Witt et al. generated an adult *Drosophila* testis atlas by scRNA-seq and identified a cluster of lineage-specific *de novo* genes, elucidating the distribution of lineage-specific *de novo* genes in spermatogenesis that might deepen our understanding of testis maintenance [49]. More importantly, further investigation indicated that the X chromosome and autosomes showed similar transcriptional activity in somatic and pre-meiotic cells, while less transcriptional activity was identified in the X chromosome than autosomes in meiotic and post-meiotic cells, implying the presence of pre-meiotic X-chromosome dosage compensation in the *Drosophila* testis [50].

Our data support the idea that the uptake of Sb added to fly food could cause concentration-dependent testicular toxicity, reducing the number of GSCs as well as clustering of elongated spermatids. Cell-cell communication among testicular components has not been extensively investigated. Furthermore, we identified multiple novel signatures highly correlated with GSCs/early spermatogonia maintenance and spermatid elongation. Among them, S-LAPs family members have been found with key roles for mitochondrial paracrystalline material in the elongated spermatids to control male fertility in *Drosophila* testes [51]. Subunits for SCP-Containing Proteins were dominant expressed in testes and involved in multicellular organism reproduction [52]. A cluster of four genes (Mst84Da, Mst84Db, Mst84Dc and Mst84Dd) for Mst84D family were also male-specifically expressed in testes and found essential roles for sperm axoneme assembly [53]. Meanwhile, seminal fluid proteins, Mst57D, and Serpin signatures secreted extracellular proteins, which were highly expressed in adult male accessory gland and transferred from males to females during mating in *Drosophila* [54, 55]. With the help of scRNA-seq technology, we have, for the first time, revealed the landscape of Sb-mediated reproductive toxicity and novel signatures at single cell resolution.

In *Drosophila*, scRNA-seq analysis of gonads allowed the reconstruction of developmental trajectories of germline cells to identify novel differentiated states [44, 49]. In our study, pseudotime trajectory analysis was used to further assess germ cell maturation and identified three novel states corresponding to two differentiative branches during spermatogenesis in *Drosophila* testes. Following Sb exposure, the number of germ cells in all three states was dramatically reduced. We also identified several novel factors and signals that were significantly altered by Sb exposure. Notably, KEGG analysis of DEGs in state 1 cells revealed that multiple metabolic processes, especially in terms of carbon metabolism, were involved in the maintenance of early stage germ cells. Importantly, in the testicular GSCs_to_Early_Spermatogonia cluster, we confirmed that carbon metabolism was highly correlated with SCP-Containing Proteins, S-LAPs, and Mst84D signatures, which are known to be specifically expressed in testes. Differential expression dynamics of lineage-specific genes will improve our understanding of differentiation stages of testicular Sb exposure-induced genetic heterogeneity.

Overall, this study provides evidence that Sb exposure negatively impacts GSC maintenance and spermatid elongation, affecting key steps of *Drosophila* spermatogenesis. This is the first study to clarify the molecular basis and regulatory signals of Sb-mediated testicular toxicity at single-cell resolution, providing new insight into the regulatory mechanism of reproductive toxicity damage caused by environmental chemicals.

## Materials and methods

### Fly stock maintenance

All flies were cultured on standard cornmeal food at 25 °C and grown at proper humidity levels. Two- to three-day-old male flies of the W^1118^ line were selected for Sb exposure and used for further functional analysis.

### Chemical exposure schedule

Potassium antimonyl tartrate trihydrate (C_8_H_4_K_2_O_12_Sb_2_·3H_2_O, 99.0–103%; Sigma-Aldrich, St. Louis, MO, USA) was used in this study. Three different environmental exposures of Sb with relevant final concentrations (0.3, 0.6, and 1.2 mg/mL) were chosen. Two- to three-day-old male flies of the W^1118^ line were then placed in standard *Drosophila* food medium containing Sb for 10 days (referred as the chemical exposed group). Control male W^1118^ flies were placed in standard *Drosophila* food medium without Sb for 10 days.

### RNA extraction, cDNA synthesis, and quantitative real-time PCR (qRT-PCR)

Total RNA was extracted using TRIzol reagent (15596026; Invitrogen, Waltham, MA, USA) from testes according to the manufacturer’s instructions. A PrimeScript™ II 1st Strand cDNA Synthesis Kit (6210 A; Takara, Shiga, Japan) was used to synthesize cDNA. qRT-PCR was performed using a LightCycler^®^ 96 Real-Time PCR System (Roche, Basel, Switzerland) using TB Green Premix Ex Taq II (RR820; Takara). Data analysis was performed using the 2^-∆∆Ct^ method. The sequences of the qRT-PCR primers are provided in Table S1.

### Immunofluorescence

Immunofluorescence was carried out as described previously [16, 23, 56]. Briefly, fly testes were dissected in 1 × phosphate-buffered saline (PBS), fixed for 30 min in 4% paraformaldehyde (PFA), washed three times with 0.3% PBS-Triton X-100 (PBST), and incubated in 5% bovine serum albumin (BSA) for 30 min. Primary antibodies were diluted in 5% BSA and testes were incubated at 25 °C for 1 h, and then washed three times with 0.3% PBST. Secondary antibodies were conjugated with Cy3 or A647 (Jackson ImmunoResearch Laboratories, West Grove, PA, USA), diluted at a ratio of 1:400 with 5% BSA, and incubated at 25 °C for 1 h avoiding light. The testes were then washed three times with 0.3% PBST and stained with Hoechst 33342 (1.0 mg/mL, C0031; Solarbio, Beijing, China), which diluted with PBS according to the potency of 1: 800, for 5 min before finalizing. Detailed information on the primary antibodies is provided in Table S2. For F-actin staining, Alexa Fluor^TM^ Plus555 Phalloidin (1:50, A30106; Invitrogen) was used according to the manufacturer’s instructions.

### Phase-contrast visualization

Testes from the flies were dissected in 1 × PBS, followed by several washes. Squashed testes on slides were coverslipped and observed under a phase-contrast microscope.

### Sample collection and processing

Two groups [Sb, 0 mg/mL (Control) and Sb, 1.2 mg/mL (Sb)] were chosen for scRNA-seq based on the above results. For each group, the testes of 180 male flies were dissected in cold PBS. The resulting mixtures of testes were washed with cold PBS three times and immediately transferred into GEXSCOPETM Tissue Preservation Solution (Singleron Biotechnologies, Cheshire, CT, USA) on ice. The pooled segments were further processed following the procedure for tissue dissociation and single-cell preparation.

### Tissue dissociation and single-cell suspension preparation

The testes were washed with Hanks Balanced Salt Solution (HBSS) three times and digested in 2 mL of GEXSCOPETM Tissue Dissociation Solution (Singleron Biotechnologies) using a Singleron PythoN™ Automated Tissue Dissociation System (Singleron Biotechnologies) at 28 °C for 15 min. The mixture was then centrifuged at 500 g for 5 min and resuspended with PBS. Finally, the samples were stained with trypan blue (Sigma-Aldrich) and cellular viability was evaluated microscopically.

### scRNA sequencing library preparation

Single-cell suspensions (1 × 10^5^ cells/mL) with PBS (HyClone, Logan, UT, USA) were loaded into microfluidic devices using a Singleron Matrix^®^ Single Cell Processing System (Singleron Biotechnologies). Subsequently, scRNA-seq libraries were constructed according to the protocol of the GEXSCOPE^®^ Single Cell RNA Library Kit (Singleron Biotechnologies) [57]. Briefly, a single-cell suspension was loaded onto the microchip to partition single cells into individual wells on the chip. Cell barcoding beads were loaded into the microchip and washed. Afterwards, 100 μL of single-cell lysis buffer was added to the chip to lyse the cells and capture mRNAs at room temperature for 20 min. The beads, together with the captured RNAs, were flushed out of the microchip and used for subsequent reverse transcription, cDNA amplification, and library construction. After size selection and purification, pools were sequenced on an Illumina Novaseq 6000 (San Diego, CA, USA) with 150 bp paired-end reads.

### scRNA-seq quantifications and statistical analysis

Raw reads were processed to generate gene expression profiles using an internal pipeline. Briefly, for each cell barcode the unique molecular identifier (UMI) was extracted after filtering read one without poly-T tails. Adapters and poly-A tails were trimmed (fastp V1) before aligning read two to GRCh38 with *Drosophila melanogaster* Ensembl version 102 annotation [58]. For reads with the same cell barcode, the UMI and gene were grouped together to calculate the number of UMIs for genes in each cell. UMI count tables for each cellular barcode were employed for further analysis. Cells with an unusually high number of UMIs (>37,000) or mitochondrial gene percent (>25%) were filtered out. We also excluded cells with less than 990 or more than 4200 genes detected.

Cell type identification and clustering analysis were performed using the Seurat program [59, 60]. Cell-by-gene matrices for each sample were individually imported to Seurat version 3.1.1 for downstream analysis [60]. Uniform manifold approximation and projection (UMAP) and t-distributed Stochastic Neighbor Embedding (t-SNE) were performed to visualize cell clusters. Upregulated enriched genes were determined to be significant with a threshold standard of fold change >1.28 and a *P*-value <0.01. Differentially expressed genes (DEGs) were considered significant with a fold change >1.50 and *P*-value <0.05.

Gene Ontology (GO) and Kyoto Encyclopedia of Genes and Genomes (KEGG) analyses were carried out on the gene set using clusterProfiler software to explore biological functions or pathways significantly associated with specifically expressed genes [61].

For correlation analysis, gene sets were calculated based on average expression counts that belong to each set of features via the PercentageFeatureSet function in the Seurat package [46]. Pearson correlations were calculated among these gene sets or signatures. Gene correlation analysis was performed directly on the data matrix by the Pearson correlation method.

Monocle 2 (version 2.10.1) was used to perform single cell trajectory analysis based on the matrix of cells and gene expression [62]. Monocle 2 reduced the space down to one with two dimensions and ordered the cells [63]. Once the cells were ordered, the trajectory was visualized in the reduced dimensional space. Pseudotime trajectory analysis was used to further analyze the germ cell differentiation trajectories to identify key factors or pathways required for different novel stages during spermatogenesis.

### Statistical analysis

Quantitative results were presented as the mean ± standard error of mean (SEM), and the data were evaluated for statistical differences using Student’s *t*-test and a one-way ANOVA with GraphPad Prism software version 6.01 (GraphPad Inc., La Jolla, CA, USA). Chi-square test was used to evaluate ratio results. Experiments conducted in this study were repeated at least three times. *P < 0.05; **P < 0.01; ***P < 0.001.

## Supplementary information

supplementary table 4

supplementary figures and table 1 and 2

supplementary table 3

## Data Availability

The raw sequence data reported in this paper have been deposited in the Genome Sequence Archive (Genomics, Proteomics & Bioinformatics 2021) in National Genomics Data Center (Nucleic Acids Res 2022), China National Center for Bioinformation / Beijing Institute of Genomics, Chinese Academy of Sciences (GSA: CRA009665) that are publicly accessible at https://ngdc.cncb.ac.cn/gsa.

## References

[1] Tylenda CA, Torres FAT, Sullivan Jr DW. Antimony. In Handbook on the Toxicology of Metals, Elsevier; 2022: 23–40.

[2] Zhang Y, Ding C, Gong D, Deng Y, Huang Y, Zheng J (2021). A review of the environmental chemical behavior, detection and treatment of antimony. Environ Technol Innov.

[3] Tao Y, Su H, Li H, Zhu Y, Shi D, Wu F (2021). Ecological and human health risk assessment of antimony (Sb) in surface and drinking water in China. J Clean Prod.

[4] Sundar S, Chakravarty J (2010). Antimony toxicity. Int J Environ Res public health.

[5] Wang X, Wang R, Zhang Z, Luo C, Zhao Z, Ruan J (2022). Level-specific associations of urinary antimony with cognitive function in US older adults from the National Health and Nutrition Examination Survey 2011–2014. BMC Geriatr.

[6] You X, Xiao Y, Liu K, Yu Y, Liu Y, Long P (2019). Association of plasma antimony concentration with markers of liver function in Chinese adults. Environ Chem.

[7] Sundar S, Sinha PR, Agrawal NK, Srivastava R, Rainey PM, Berman JD (1998). A cluster of cases of severe cardiotoxicity among kala-azar patients treated with a high-osmolarity lot of sodium antimony gluconate. Am J Trop Med Hyg.

[8] Jiang J, Wu Y, Sun G, Zhang L, Li Z, Sommar J (2021). Characteristics, accumulation, and potential health risks of antimony in atmospheric particulate matter. ACS Omega.

[9] Xu S, Yang Z, Zhi Y, Yu S, Zhang T, Jiang J (2021). The effects of antimony on Alzheimer’s disease-like pathological changes in mice brain. Sci Total Environ.

[10] Yu S, Li Z, Zhang Q, Wang R, Zhao Z, Ding W (2022). GPX4 degradation via chaperone-mediated autophagy contributes to antimony-triggered neuronal ferroptosis. Ecotoxicol Environ Saf.

[11] Kumar N, Singh AK (2022). Impact of environmental factors on human semen quality and male fertility: a narrative review. Environ Sci Eur.

[12] Wu S, Zhong G, Wan F, Jiang X, Tang Z, Hu T (2021). Evaluation of toxic effects induced by arsenic trioxide or/and antimony on autophagy and apoptosis in testis of adult mice. Environ Sci Pollut Res Int.

[13] Omura M, Tanaka A, Hirata M, Inoue N (2002). Testicular toxicity evaluation of two antimony compounds, antimony trioxide and antimony potassium tartrate, in rats and mice. Environ Health Prev Med.

[14] Demir E, Turna Demir F (2023). *Drosophila* melanogaster as a dynamic in vivo model organism reveals the hidden effects of interactions between microplastic/nanoplastic and heavy metals. J Appl Toxicol.

[15] Bellen HJ, Tong C, Tsuda H (2010). 100 years of *Drosophila* research and its impact on vertebrate neuroscience: a history lesson for the future. Nat Rev Neurosci.

[16] Yu J, Zheng Q, Li Z, Wu Y, Fu Y, Wu X (2021). CG6015 controls spermatogonia transit-amplifying divisions by epidermal growth factor receptor signaling in *Drosophila* testes. Cell Death Dis.

[17] White-Cooper H (2010). Molecular mechanisms of gene regulation during *Drosophila* spermatogenesis. Reproduction.

[18] Barreau C, Benson E, Gudmannsdottir E, Newton F, White-Cooper H (2008). Post-meiotic transcription in *Drosophila* testes. Development.

[19] Xu R, Li J, Zhao H, Kong R, Wei M, Shi L (2018). Self-restrained regulation of stem cell niche activity by niche components in the *Drosophila* testis. Dev Biol.

[20] Carbonell A, Pérez-Montero S, Climent-Cantó P, Reina O, Azorín F (2017). The germline linker histone dBigH1 and the translational regulator bam form a repressor loop essential for male germ stem cell differentiation. Cell Rep.

[21] Chang YC, Tu H, Chen J-Y, Chang C-C, Yang SY, Pi H (2019). Reproduction disrupts stem cell homeostasis in testes of aged male *Drosophila* via an induced microenvironment. PLoS Genet.

[22] Yu J, Lan X, Chen X, Yu C, Xu Y, Liu Y (2016). Protein synthesis and degradation are essential to regulate germline stem cell homeostasis in *Drosophila* testes. Development.

[23] Li Z, Wu Y, Fu Y, Chen X, Zhao X, Wu X (2022). Cyst stem cell lineage eIF5 non-autonomously prevents testicular germ cell tumor formation via eIF1A/eIF2γ-mediated pre-initiation complex. Stem Cell Res Ther.

[24] Amoyel M, Anderson J, Suisse A, Glasner J, Bach EA (2016). Socs36E controls niche competition by repressing MAPK signaling in the *Drosophila* testis. PLoS Genet.

[25] Demarco RS, Eikenes ÅH, Haglund K, Jones DL (2014). Investigating spermatogenesis in *Drosophila* melanogaster. Methods.

[26] Nandi A, Chowdhuri DK (2021). Cadmium mediated redox modulation in germline stem cells homeostasis affects reproductive health of *Drosophila* males. J Hazard Mater.

[27] Sênos Demarco R, Stack BJ, Tang AM, Voog J, Sandall SL, Southall TD (2022). Escargot controls somatic stem cell maintenance through the attenuation of the insulin receptor pathway in *Drosophila*. Cell Rep.

[28] Insco ML, Leon A, Tam CH, McKearin DM, Fuller MT (2009). Accumulation of a differentiation regulator specifies transit amplifying division number in an adult stem cell lineage. Proc Natl Acad Sci USA.

[29] Li Y, Minor NT, Park JK, McKearin DM, Maines JZ (2009). Bam and Bgcn antagonize Nanos-dependent germ-line stem cell maintenance. Proc Natl Acad Sci USA.

[30] Hwa JJ, Hiller MA, Fuller MT, Santel A (2002). Differential expression of the *Drosophila* mitofusin genes fuzzy onions (fzo) and dmfn. Mech Dev.

[31] Baker CC, Fuller MT (2007). Translational control of meiotic cell cycle progression and spermatid differentiation in male germ cells by a novel eIF4G homolog. Development.

[32] Xu S, Hafer N, Agunwamba B, Schedl P (2012). The CPEB protein Orb2 has multiple functions during spermatogenesis in *Drosophila* melanogaster. PLoS Genet.

[33] Yu J, Luan X, Yan Y, Qiao C, Liu Y, Zhao D (2019). Small ribonucleoprotein particle protein SmD3 governs the homeostasis of germline stem cells and the crosstalk between the spliceosome and ribosome signals in *Drosophila*. FASEB J.

[34] Li H, Janssens J, De Waegeneer M, Kolluru SS, Davie K, Gardeux V (2022). Fly cell atlas: a single-nucleus transcriptomic atlas of the adult fruit fly. Science.

[35] Micchelli CA, Perrimon N (2006). Evidence that stem cells reside in the adult *Drosophila* midgut epithelium. Nature.

[36] Ohlstein B, Spradling A (2006). The adult *Drosophila* posterior midgut is maintained by pluripotent stem cells. Nature.

[37] Jasper H (2020). Intestinal stem cell aging: origins and interventions. Annu Rev Physiol.

[38] Shi J, Fok KL, Dai P, Qiao F, Zhang M, Liu H (2021). Spatio-temporal landscape of mouse epididymal cells and specific mitochondria-rich segments defined by large-scale single-cell RNA-seq. Cell Disco.

[39] Kan T, Zhang S, Zhou S, Zhang Y, Zhao Y, Gao Y (2022). Single-cell RNA-seq recognized the initiator of epithelial ovarian cancer recurrence. Oncogene.

[40] Li H (2021). Single-cell RNA sequencing in *Drosophila*: technologies and applications. Wiley Interdiscip Rev Dev Biol.

[41] Allen AM, Neville MC, Birtles S, Croset V, Treiber CD, Waddell S (2020). A single-cell transcriptomic atlas of the adult *Drosophila* ventral nerve cord. eLife.

[42] Cattenoz PB, Sakr R, Pavlidaki A, Delaporte C, Riba A, Molina N (2020). Temporal specificity and heterogeneity of *Drosophila* immune cells. EMBO J.

[43] Hung R-J, Hu Y, Kirchner R, Liu Y, Xu C, Comjean A (2020). A cell atlas of the adult *Drosophila* midgut. Proc Natl Acad Sci USA.

[44] Jevitt A, Chatterjee D, Xie G, Wang X-F, Otwell T, Huang Y-C (2020). A single-cell atlas of adult *Drosophila* ovary identifies transcriptional programs and somatic cell lineage regulating oogenesis. PLoS Biol.

[45] Tiwari AK, Pragya P, Ravi Ram K, Chowdhuri DK (2011). Environmental chemical mediated male reproductive toxicity: *Drosophila* melanogaster as an alternate animal model. Theriogenology.

[46] Li Y, Mi P, Wu J, Tang Y, Liu X, Cheng J (2022). High throughput scRNA-Seq provides insights Into leydig cell senescence induced by experimental autoimmune orchitis: a prominent role of interstitial fibrosis and complement activation. Front Immunol.

[47] Han X, Wang R, Zhou Y, Fei L, Sun H, Lai S (2018). Mapping the mouse cell atlas by microwell-seq. Cell.

[48] Zhao L, Yao C, Xing X, Jing T, Li P, Zhu Z (2020). Single-cell analysis of developing and azoospermia human testicles reveals central role of Sertoli cells. Nat Commun.

[49] Witt E, Benjamin S, Svetec N, Zhao L (2019). Testis single-cell RNA-seq reveals the dynamics of de novo gene transcription and germline mutational bias in *Drosophila*. eLife.

[50] Witt E, Shao Z, Hu C, Krause HM, Zhao L (2021). Single-cell RNA-sequencing reveals pre-meiotic X-chromosome dosage compensation in *Drosophila* testis. PLoS Genet.

[51] Laurinyecz B, Vedelek V, Kovács AL, Szilasi K, Lipinszki Z, Slezák C (2019). Sperm-Leucylaminopeptidases are required for male fertility as structural components of mitochondrial paracrystalline material in *Drosophila* melanogaster sperm. PLoS Genet.

[52] Gaudet P, Livstone MS, Lewis SE, Thomas PD (2011). Phylogenetic-based propagation of functional annotations within the Gene Ontology consortium. Brief Bioinform.

[53] Kuhn R, Kuhn C, Börsch D, Glätzer KH, Schäfer U, Schäfer M (1991). A cluster of four genes selectively expressed in the male germ line of *Drosophila* melanogaster. Mech Dev.

[54] Findlay GD, Yi X, Maccoss MJ, Swanson WJ (2008). Proteomics reveals novel *Drosophila* seminal fluid proteins transferred at mating. PLoS Biol.

[55] Findlay GD, MacCoss MJ, Swanson WJ (2009). Proteomic discovery of previously unannotated, rapidly evolving seminal fluid genes in *Drosophila*. Genome Res.

[56] Zheng Q, Chen X, Qiao C, Wang M, Chen W, Luan X (2021). Somatic CG6015 mediates cyst stem cell maintenance and germline stem cell differentiation via EGFR signaling in *Drosophila* testes. Cell Death Disco.

[57] Dura B, Choi J-Y, Zhang K, Damsky W, Thakral D, Bosenberg M (2019). scFTD-seq: freeze-thaw lysis based, portable approach toward highly distributed single-cell 3’ mRNA profiling. Nucleic Acids Res.

[58] Liao Y, Smyth GK, Shi W (2014). featureCounts: an efficient general purpose program for assigning sequence reads to genomic features. Bioinformatics.

[59] Satija R, Farrell JA, Gennert D, Schier AF, Regev A (2015). Spatial reconstruction of single-cell gene expression data. Nat Biotechnol.

[60] Butler A, Hoffman P, Smibert P, Papalexi E, Satija R (2018). Integrating single-cell transcriptomic data across different conditions, technologies, and species. Nat Biotechnol.

[61] Yu G, Wang L-G, Han Y, He Q-Y (2012). clusterProfiler: an R package for comparing biological themes among gene clusters. OMICS.

[62] Trapnell C, Cacchiarelli D, Grimsby J, Pokharel P, Li S, Morse M (2014). The dynamics and regulators of cell fate decisions are revealed by pseudotemporal ordering of single cells. Nat Biotechnol.

[63] Qiu X, Mao Q, Tang Y, Wang L, Chawla R, Pliner HA (2017). Reversed graph embedding resolves complex single-cell trajectories. Nat Methods.

