# Supplementary material for: Single-cell RNA sequencing reveals cell landscape following antimony exposure during spermatogenesis in Drosophila testes
Source: Cell Death Discov. 2023 Mar 9;9:86. doi: 10.1038/s41420-023-01391-4 (PMC9998446; doi:10.1038/s41420-023-01391-4)
Supplement: Supplementary file 2 — supplementary figures and table 1 and 2 [file 41420_2023_1391_MOESM2_ESM.docx]

Supplementary Materials for

**Single-cell RNA sequencing reveals cell landscape following antimony exposure during spermatogenesis in *Drosophila* testes**

**
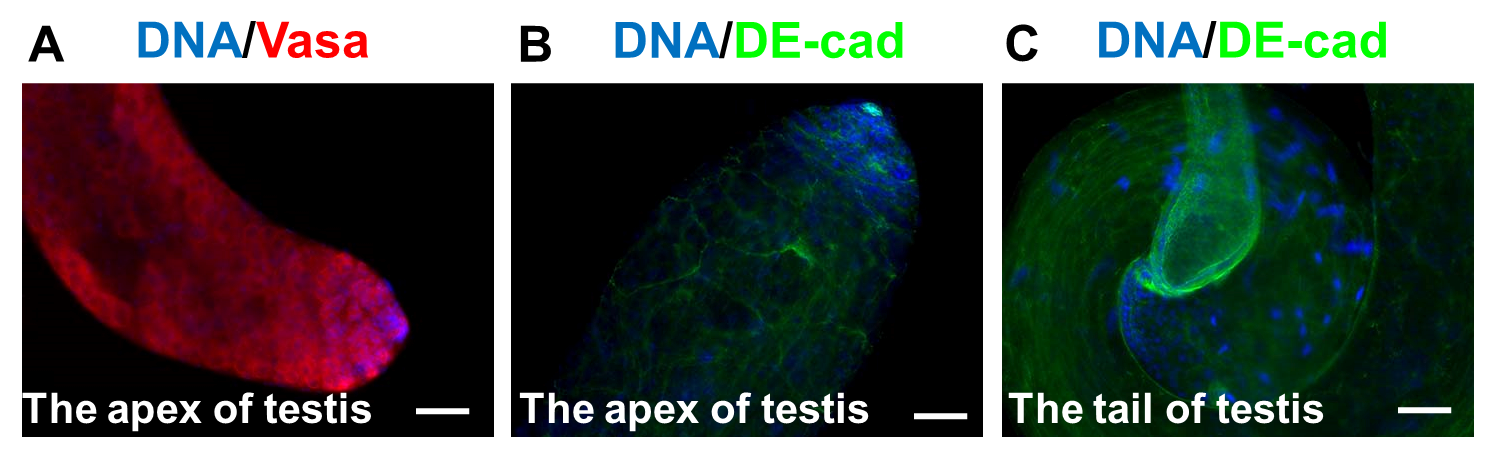
**

**Figure S1. Testicular immunostainings in *Drosophila*.** (A) Immunostaining of Vasa (red) at the apex of testis. (B) Immunostaining of DE-cad (green) at the apex of testis. (C) Immunostaining of DE-cad (green) at the tail of testis. DNA was stained with Hoechst33342 (blue). Scale bar: 50 μm.


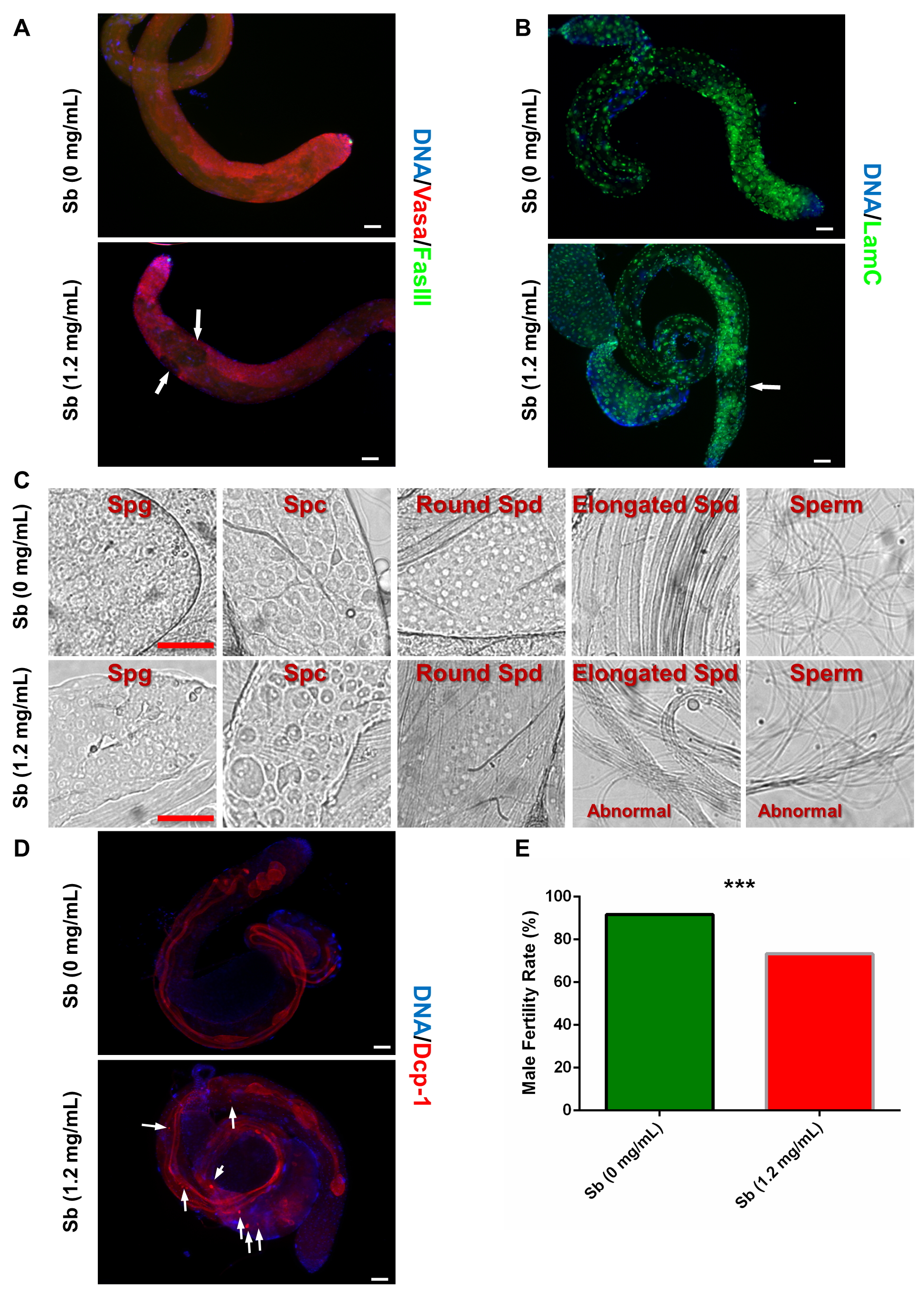


**Figure S2. Testicular phenotypic analysis of Sb exposure in *Drosophila*.** (A) Whole mount immunostaining of Vasa (red) and FasIII (green) in control (Sb, 0 mg/mL) and Sb (Sb, 1.2 mg/mL) groups. Representative missing germ cell cysts were marked with white arrows. (B) Immunostaining of testicular LamC (green) in control (Sb, 0 mg/mL) and Sb (Sb, 1.2 mg/mL) groups. Representative missing spermatocyte cyst was marked with the white arrow. (C) Phase-contrast view of testes in control (Sb, 0 mg/mL) and Sb (Sb, 1.2 mg/mL) groups. Illustrations are the different stages of spermatogenesis, including spermatogonia (Spg), spermatocytes (Spc), round spermatids (Round Spd), elongated spermatids (Elongated Spd) and mature sperm (Sperm). Abnormal Elongated Spd and Sperm were shown in Sb (Sb, 1.2 mg/mL) group. (D) Immunostaining of Dcp-1 (red) of testes in control (Sb, 0 mg/mL) and Sb (Sb, 1.2 mg/mL) groups. Representative Dcp-1 positive apoptotic cells were marked with white arrows. (E) Male fertility rate (%) in control (Sb, 0 mg/mL; n = 12) and Sb (Sb, 1.2 mg/mL; n = 30) groups. DNA was stained with Hoechst33342 (blue). Scale bar: 50 μm. ****P* < 0.001.


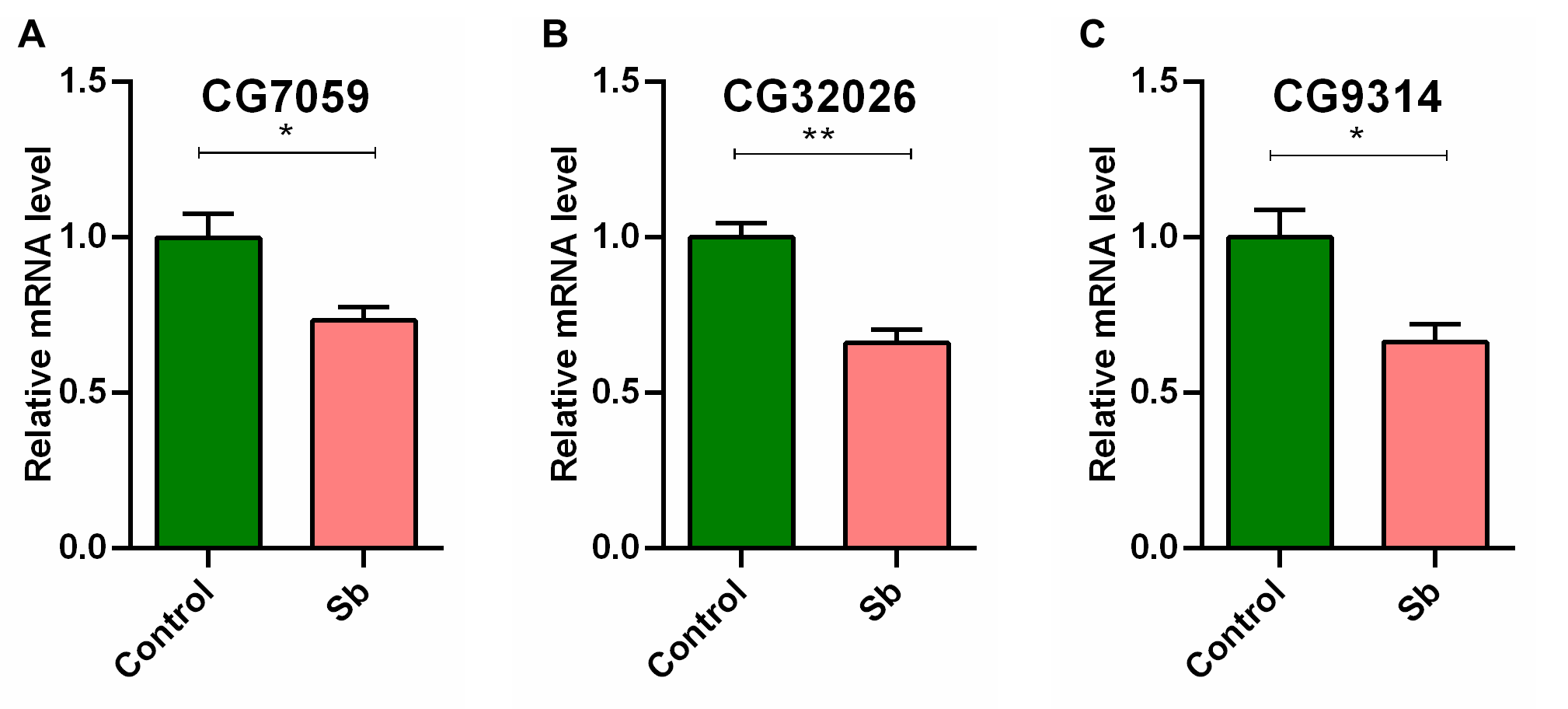


**Figure S3. The relative mRNA levels of representative carbon metabolism related DEGs in *Drosophila* testes.** qRT-PCR analysis of *CG7059* (A), *CG32026* (B), and *CG9314* (C) for carbon metabolism pathway in control and Sb groups. **P* < 0.05, ***P* < 0.01.


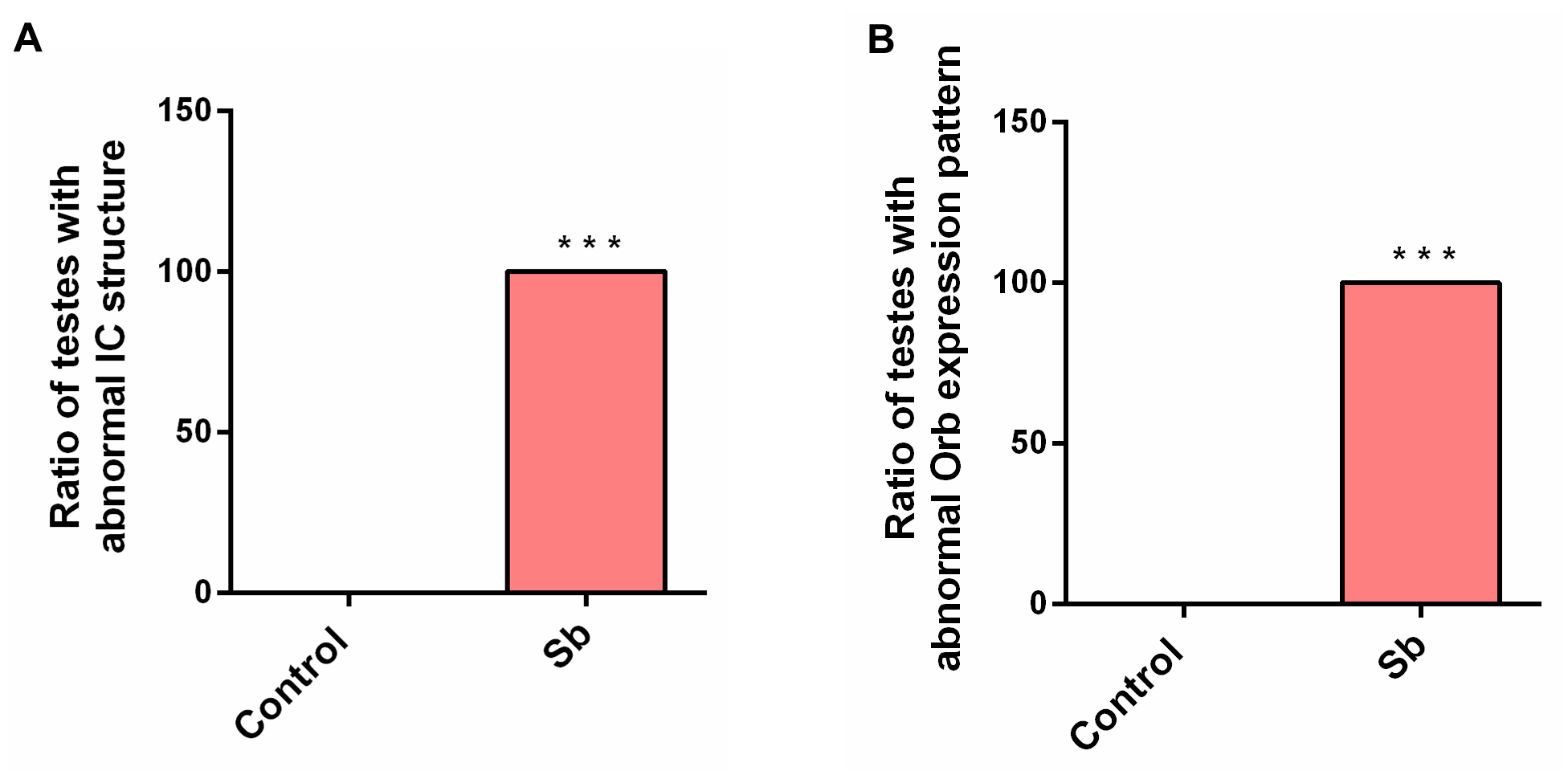


**Figure S4. Quantification F-actin and Orb.** (A) Ratio of testes with abnormal IC structure in control (n = 7) and Sb (n = 8) groups. (B) Ratio of testes with abnormal Orb expression pattern in control (n = 5) and Sb (n = 6) groups. ****P* < 0.001.

**Table S1. Detailed primer information used for qRT-PCR analysis.**

| **Gene** | **Forward (5'-3')** | **Reverse (5'-3')** |
| --- | --- | --- |
| scpr-A | ACTTCGTACTCACCTGCAACTTCG | CTCCTTGGCGTAGCACAGATTGG |
| scpr-B | AGCCGAGACAGAGTACACCGATG | AACTTGGTCACCGCCTTGTTGG |
| scpr-C | CAACCTGTGCTACGCCAAGGAG | GTCCATTCCATGCCTACATCGTCTG |
| S-Lap1 | GCCGACGATATGTCCCGAAGTTC | GTTCTTGTCCTCCTCATCCGCATAG |
| S-Lap3 | CGAGAATCTGCCGTCTGGAATGTC | ATCCGAGATGACCACCACTCCTG |
| Mst84Dc | ATCGTGCTGCGGATATTACTGCTG | ACAAGGTCCACAGGGTCCACAG |
| Mst57Da | CTTTGCTGGTTGTTCTGGCTTTGG | CTGGCTTCTTGGCACCGATGAC |
| Mst57Db | GCCGACATGAAGATCACCTCTGC | GCGCTGGGACTATTGATGTGGATC |
| Mst57Dc | TGTGTGGAGTCCTGGGATCTAACG | CAGTTAGGCGGCAAGGTTAAAATCC |
| Spn28F | TCGCTTTGCCGACGATTTCTACC | TCCTCATTTCTTGGGCTGTCTTTGC |
| Spn38F | GCTTCACCGACGATCTCTACCAAC | ATGCCAGGCTCAGTGCGATTTC |
| Sfp65A | GACTTACGGAAGCGGAGGGAAATAC | GATCTGGCAATCGAGGAGATGAACC |
| Sfp70A4 | GGAATGGTTGAGGCTCGAAGAAGG | ATATCTGGTTCCTGGTGGATTGTGC |
| CG7059 | ATTGGCGGCTGTGCGAAAGG | CCTGAACCTGCTCCTCCCCATAG |
| CG32026 | AAAGAGAGAAATCCCAGTGCGACAG | GTGGCGGTTGAATGCGAGAGG |
| CG9314 | TCTGGACCTACACCCTGGATAACTG | CCTCCGTCATCAGTCTTCCGAAATC |
| Gapdh | GTGGTGAACGGCCAGAAGAT | GCCTTGTCAATGGTGGTGAA |

**Table S2.Antibodies used in this study.**

| **Antigen** | **Source** | **Company** | **Catalog** | **Application** | **Dilution** |
| --- | --- | --- | --- | --- | --- |
| Vasa | Rabbit | A gift from Prof. Chao Tong | N/A | IF | 1:1000 |
| Orb | Mouse | DSHB | 4H8 | IF | 1:50 |
| DE-cad | Rat | DSHB | DCAD2 | IF | 1:15 |
| FasⅢ | Mouse | DSHB | 7G10 | IF | 1:30 |
| LamC | Mouse | DSHB | LC28.26 | IF | 1:100 |
| Dcp-1 | Rabbit | CST | #9578 | IF | 1:200 |
